# Calcimimetics Alter Periosteal and Perilacunar Bone Matrix Composition and Material Properties in Early Chronic Kidney Disease

**DOI:** 10.1002/jbmr.4574

**Published:** 2022-06-03

**Authors:** John G. Damrath, Sharon M. Moe, Joseph M. Wallace

**Affiliations:** ^1^ Weldon School of Biomedical Engineering Purdue University West Lafayette IN USA; ^2^ Department of Medicine, Division of Nephrology Indiana University School of Medicine Indianapolis IN USA; ^3^ Department of Biomedical Engineering Indiana University‐Purdue University at Indianapolis Indianapolis IN USA

**Keywords:** CHRONIC KIDNEY DISEASE‐MINERAL AND BONE DISORDER, CALCIMIMETICS, BONE QUALITY, RAMAN SPECTROSCOPY, NANOINDENTATION, PERILACUNAR

## Abstract

Chronic kidney disease (CKD) affects 15% of Americans and greatly increases fracture risk due to elevated parathyroid hormone, cortical porosity, and reduced bone material quality. Calcimimetic drugs are used to lower parathyroid hormone (PTH) in CKD patients, but their impact on bone matrix properties remains unknown. We hypothesized that tissue‐level bone quality is altered in early CKD and that calcimimetic treatment will prevent these alterations. To test this hypothesis, we treated Cy/+ rats, a model of spontaneous and progressive CKD‐mineral and bone disorder (CKD‐MBD), with KP‐2326, a preclinical analogue of etelcalcetide, early in the CKD disease course. To measure tissue‐level bone matrix composition and material properties, we performed colocalized Raman spectroscopy and nanoindentation on new periosteal bone and perilacunar bone using hydrated femur sections. We found that CKD and KP treatment lowered mineral type B carbonate substitution whereas KP treatment increased mineral crystallinity in new periosteal bone. Reduced elastic modulus was lower in CKD but was not different in KP‐treated rats versus CTRL. In perilacunar bone, KP treatment lowered type B carbonate substitution, increased crystallinity, and increased mineral‐to‐matrix ratio in a spatially dependent manner. KP treatment also increased reduced elastic modulus and hardness in a spatially dependent manner. Taken together, these data suggest that KP treatment improves material properties on the tissue level through a combination of lowering carbonate substitution, increasing mineral crystallinity, and increasing relative mineralization of the bone early in CKD. As a result, the mechanical properties were improved, and in some regions, were the same as control animals. Therefore, calcimimetics may help prevent CKD‐induced bone deterioration by improving bone quality in new periosteal bone and in bone tissue near osteocyte lacunae. © 2022 The Authors. *Journal of Bone and Mineral Research* published by Wiley Periodicals LLC on behalf of American Society for Bone and Mineral Research (ASBMR).

## Introduction

Chronic kidney disease (CKD) affects 15% of Americans and results in long‐term mineral dysregulation, increasing the risk of skeletal fractures by fourfold relative to the age‐ and sex‐matched healthy population.^(^ ^1^, ^2^ ^)^ Skeletal fragility in CKD is precipitated by a combination of biochemical alterations including reduced 1,25‐dihydroxyvitamin D and elevated parathyroid hormone (PTH).^(^ ^3^ ^)^ PTH, a potent regulator of serum calcium, has been positively correlated with the formation of pores in the cortical bone compartment, reducing its ability to bear loads on the structural level.^(^ ^4^ ^)^ Although cortical porosity is typically observed late in CKD, fracture risk is elevated across all stages of CKD.^(^ ^1^ ^)^ Taken together, these findings indicate that CKD has a detrimental impact on bone matrix quality, increasing skeletal fragility on the intrinsic material level. Importantly, fracture‐related mortality is up to three times higher in dialysis patients versus the age‐ and sex‐matched population, indicating not only the need for therapies to reduce fractures, but to develop strategies to prevent fractures earlier in CKD.^(^ ^5^ ^)^

Calcimimetics, a class of calcium‐sensing receptor allosteric agonists, are frequently used to lower PTH levels in patients on dialysis. Previously, a secondary analysis of the Evaluation of Cinacalcet Hydrochloride Therapy to Lower Cardiovascular Events (EVOLVE) trial demonstrated that calcimimetics may lower fracture incidence in patients with CKD, although the mechanism underlying this improvement has not been explored in clinical data.^(^ ^6^ ^)^ Lowering PTH has been shown to cause cortical pore infilling in preclinical models of CKD and may therefore improve bone mineral density (BMD).^(^ ^7^ ^)^ Although BMD is becoming more widely used to assess bone health in patients with CKD, high mineral content and bone volume do not singularly improve bone toughness, the ability for the tissue to dissipate energy and prevent microdamage from rapidly progressing to mechanical failure.^(^ ^8^ ^)^ Therefore, calcimimetics may partially increase fracture resistance by improving bone quality.

Matrix quality refers to the structural and compositional state of bone tissue on the nanoscales and microscales, understanding how these characteristics translate into material properties that impact whole‐bone mechanical integrity, and is critical to assessing fracture risk. Techniques such as Fourier‐transform infrared spectroscopy (FTIR) and Raman spectroscopy have become widely utilized to measure compositional bone quality, including relative mineralization and crystallinity, and can be performed with a ~1‐μm resolution.^(^ ^9^, ^10^ ^)^ Nanoindentation has become more common to measure intrinsic tissue stiffness and hardness on a similar length scale.^(^ ^11^ ^)^ Although these techniques provide a glimpse of the true tissue‐level properties of bone, the heterogeneity of tissue properties at this scale can become problematic when averaging large datasets together without controlling for the age of the tissue in a specific region of bone and how recently it was formed, as newly formed bone will have lower crystallinity.^(^ ^12^ ^)^ Therefore, methods to control for tissue age are necessary, especially in the context of CKD where a progressive loss of kidney function is linked to worsened skeletal properties over time.

The mixed anabolic and catabolic effects of PTH on the skeleton are largely attributed to PTH signaling in osteocytes. Osteocytes respond to PTH by lowering sclerostin expression and secretion, increasing osteoblast bone formation activity.^(^ ^13^ ^)^ When chronically elevated, as seen in CKD, PTH increases osteocyte‐derived receptor activator of nuclear kappa‐B ligand (RANKL), leading to increased osteoclast differentiation and bone resorption.^(^ ^14^ ^)^ In rodents, these bone formation and resorption events primarily occur near bone surfaces, making the periosteal surface a promising location to detect matrix changes in CKD with high PTH and following calcimimetic treatment. Perilacunar remodeling, the process by which osteocytes alter their surrounding bone matrix, is also driven by PTH signaling.^(^ ^15^, ^16^ ^)^ This process involves a shift toward an osteoclastic gene expression profile within the osteocyte, including higher tartrate‐resistant acid phosphatase, membrane‐bound proton pumps, matrix metalloproteinases, and cathepsin K, indicating that osteocytes are poised to remove both mineral and collagen.^(^ ^17^, ^18^ ^)^ Although fewer studies have examined osteocytic matrix modeling, osteocytes upregulate mineral deposition genes in response to anabolic stimuli such as mechanical loading, and type I collagen expression has been detected using in vitro two‐dimensional (2D) osteocyte culture models.^(^ ^19^ ^)^ Further, PTH signaling is essential for perilacunar remodeling and has been shown to mediate extracellular acidification, an essential step in the removal of bone mineral in the perilacunar region.^(^ ^20^ ^)^ Therefore, it is plausible that the bone matrix near the osteocyte lacunae may present with differential compositional and mechanical properties in the settings of high PTH and calcimimetic‐induced PTH suppression.

Previous studies from our group have demonstrated that the male Cy/+ rat, a model of progressive CKD‐mineral and bone disorder (CKD‐MBD), robustly develops hyperparathyroidism and cortical porosity by 35 weeks of age.^(^ ^21^ ^)^ We recently noted that at 28 weeks of age, the CKD rats display hyperparathyroidism without altered cortical bone geometry or increased porosity,^(^ ^22^ ^)^ making this a potentially valuable time point to examine whether tissue‐level composition and material properties are altered prior to cortical pore formation. Furthermore, this early time point provides the opportunity to determine whether calcimimetic treatment can prevent or limit CKD‐induced alterations in bone quality. We hypothesized that bone matrix composition is disrupted and material‐level mechanical properties are reduced in early CKD prior to the onset of structural deficits such as increased cortical porosity, and that calcimimetics will prevent these deleterious alterations. To test this hypothesis, we utilized male Cy/+ rats aged to 28 weeks, representing stage 4 CKD (~30% kidney function), and compared them to their healthy littermates and CKD rats treated with a calcimimetic. We herein describe a novel method to evaluate colocalized compositional and material properties in the native hydrated state in newly formed periosteal bone guided by fluorochrome labeling, and in the osteocyte perilacunar region.

## Materials and Methods

### Animal model

The Cy/+ rat model, containing a mutation in *Anks6* or SamCystin on a Sprague Dawley background, was utilized as a gradually progressive model of CKD‐MBD. Male, but not female, Cy/+ rats develop end‐stage kidney disease (ESKD) by 35 weeks of age. Previously, our group demonstrated that Cy/+ rats have elevated PTH without increased cortical porosity at 28 weeks of age.^(^ ^22^ ^)^ We utilized animals from a previous study designed to evaluate CKD‐MBD to determine whether matrix‐level bone quality is altered early in CKD.^(^ ^22^ ^)^ Three groups of 28‐week‐old male Cy/+ rats were used (*n* = 12 per group):

1) Control littermates (CTRL)
2) Untreated Cy/+ rats (CKD)
3) Cy/+ rats treated with the calcimimetic drug KP‐2326 (CKD/KP)

**Fig. 1:**
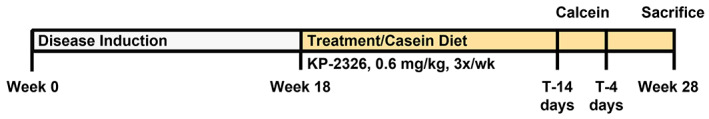
Study timeline. Animals were divided into three groups: Control littermates (CTRL), Cy/+ (CKD), and KP‐treated Cy/+ (CKD/KP). All animals were switched to a casein‐based diet at 18 weeks of age. At this time, CKD/KP animals began treatment with 0.6 mg/kg KP‐2326, thrice weekly. Calcein fluorochrome labels were administered 4 and 14 days prior to euthanasia at 28 weeks of age.

The study timeline is summarized in Fig. 1. All groups were placed on a standard rodent chow until 18 weeks of age, after which the animals were switched to a casein‐based diet (0.7% Pi, 0.6% Ca; Teklad, Madison, WI, USA; Envigo Teklad TD.04539). The casein diet increases dietary phosphorus content and creates a consistent CKD‐MBD‐like phenotype in CKD rats but does not alter biochemistries, vasculature, or bone in CTRL rats.^(^ ^21^ ^)^ Treatment with KP‐2326, a preclinical form of etelcalcetide, began at 18 weeks of age (0.6 mg/kg, thrice weekly, intraperitoneal injection [i.p.] in phosphate‐buffered saline [PBS]) with the dose determined by pilot study to identify the most potent decrease in PTH without significant hypocalcemia. In our preliminary studies, this dose was shown to lower PTH without altering serum calcium levels.^(^ ^22^ ^)^ Calcein (30 mg/kg, i.p.) was administered 4 and 14 days prior to the end of the study to label actively forming bone surfaces. At 28 weeks of age, the animals were euthanized by cardiac puncture and exsanguination followed by bilateral pneumothorax. The left femurs were then harvested, wrapped in PBS‐soaked gauze, and stored at −20°C until ready for sample preparation. All animal protocols were approved by the Indiana University‐Purdue University at Indianapolis Animal Care and Use Committee.

### Sample preparation

**Fig. 2:**
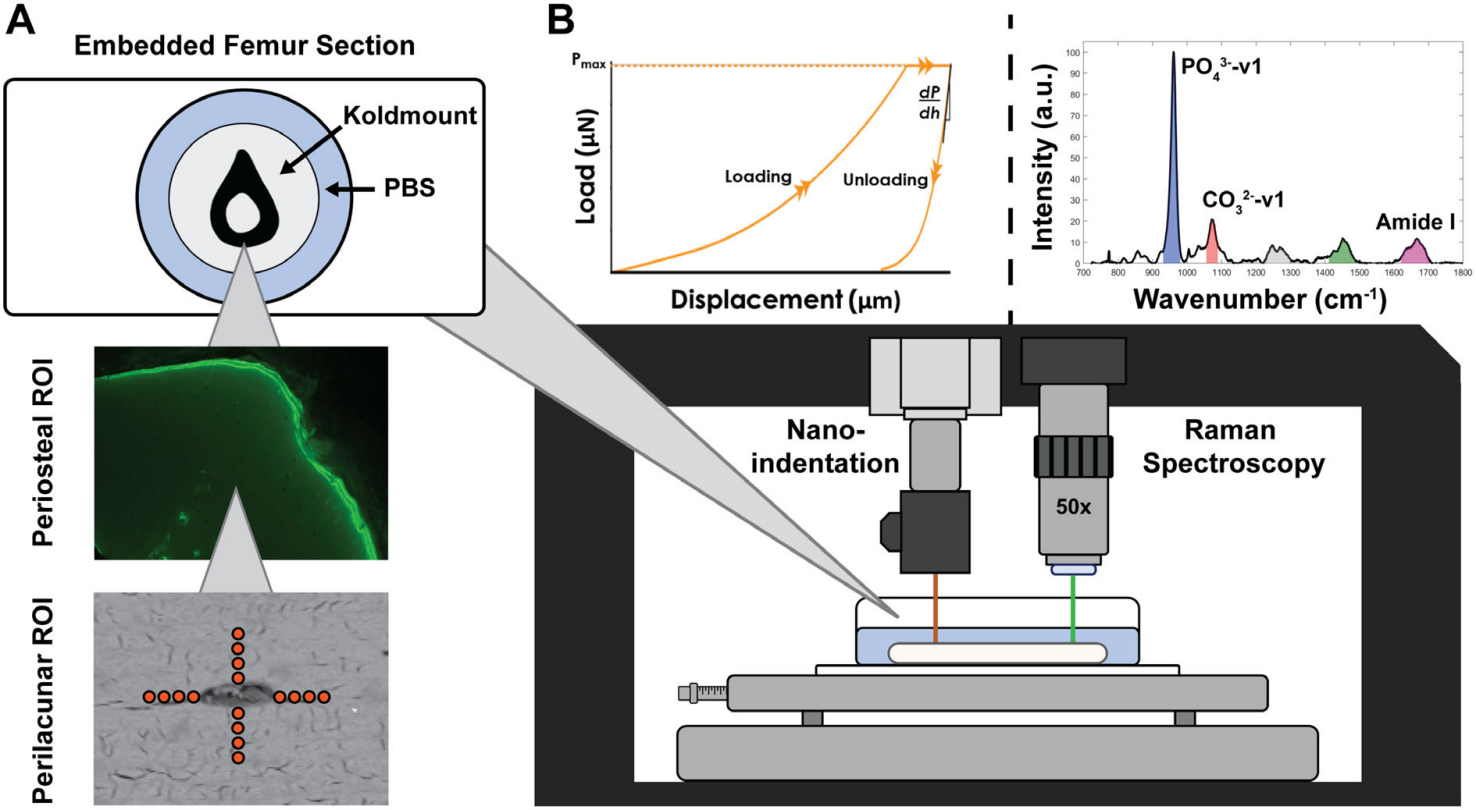
Left femurs were embedded in Koldmount, sectioned, sanded, polished, and sonicated. To prepare for colocalized Raman spectroscopy and nanoindentation, a Petri dish was Superglued to a glass slide and the embedded sections were Superglued into the dish (*A*, upper). Fluorochrome labels were used to select age‐matched periosteal regions (*A*, middle), while a second section was used to select perilacunar regions 1, 3, 5, and 7 μm from the lacunar wall of six lacunae per sample (*A*, lower). The hybrid system chamber (*B*, lower) was humidified prior to performing all Raman spectroscopy, which was analyzed for phosphate, carbonate, and amide I bands (*B*, upper right). PBS was added to the Petri dish to submerge the samples prior to nanoindentation. P_max_ and the slope of the unloading curve (dP/dh) were used to calculate material properties at the same locations where Raman was performed (*B*, upper left). dP/dh = slope of the unloading curve; PBS = phosphate‐buffered saline.

The methods for sample preparation and subsequent analyses are summarized in Fig. 2. Left femurs were placed into borosilicate glass culture tubes with the proximal end down. The proximal end was stabilized in modeling clay such that the sides of the bone were not touching the sides of the tube. Femurs were embedded with a rapid‐curing methylmethacrylate resin (Koldmount; Sigma‐Aldrich, St. Louis, MO, USA), providing stability for subsequent polishing without infiltrating the bone matrix or dehydrating the specimen. After curing, two 1.5‐mm‐thick sections were cut beginning at 37% along the length of the bone from the proximal end. Sections were sanded on both sides to 1 mm thickness. Specimens were polished on each side using first a 3 μm and then a 0.05 μm water‐based diamond suspension (Buehler, Lake Bluff, IL, USA). Finally, specimens were sonicated in PBS for 1 minute to remove residual polishing debris. Fluorescent imaging was performed to visualize calcein labels, marking regions of newly formed bone on the periosteal surface (Fig. 2*A*). These regions were mapped and utilized for selecting points in subsequent Raman spectroscopy, nanoindentation, and quantitative backscatter electron imaging.

### Colocalized Raman spectroscopy and nanoindentation

Raman spectroscopy (inVia confocal Raman microscope; Renishaw, Wotton‐under‐Edge, United Kingdom) and nanoindentation (Hysitron TI 980 TriboIndenter; Bruker, Billerica, MA, USA) were performed using a custom hybrid system equipped with a 50× objective, 785‐nm laser and a 1.03‐μm radius spherical diamond probe. Just prior to measurements, the samples were Superglued to the bottom of a Petri dish which was then Superglued to a glass slide. The glass slide maintains rigid contact without slipping using a vacuum pump fed into the stage of the hybrid system. Prior to placing the samples, the interior chamber was humidified with damp paper towels to minimize sample dehydration and fluid evaporation. Submerging the samples in PBS was not possible at this step because it distorts the laser path, greatly lowering the amount of detectable Raman scattering from the bone. Raman spectroscopy was performed at eight locations between calcein labels to analyze the matrix composition of newly formed bone and to control for tissue age across all specimens. Spectrographs were averaged across eight accumulations with 8 seconds of exposure at 50% laser power, with a spatial resolution of ~1 μm. The averaged spectrograph underwent baseline correction using an 11th order polynomial followed by cosmic ray removal. Smoothing was performed using a modified Savitzky‐Golay function with maximum likelihood estimation to preserve features near bands of interest. Compositional parameters were calculated from smoothed spectrographs as described previously: mineral‐to‐matrix ratio (MMR, ν1‐PO_4_^3−^/Amide I band areas), type B carbonate substitution (ν1‐CO_3_^2−^/ν1‐PO_4_^3−^ band areas), and crystallinity (inverse of the full width at half maximum of a Gaussian curve fitted to ν1‐PO_4_^3−^) using a custom MATLAB script (MathWorks, Natick, MA, USA) (Fig. 2*B*, upper right).^(^ ^23^ ^)^ The output parameters from each specimen were averaged, yielding one value per parameter per specimen.

Immediately following Raman spectroscopy, nanoindentation was performed at the same locations to determine material properties with a 1‐μm resolution (Fig. 2*B*, upper left). The dish containing the sample was filled with PBS to maintain hydration throughout the course of indentation. Indents were performed in load control up to 1000 μN, held for 45 seconds, and subsequently unloaded. The resulting load–displacement curves were analyzed for reduced elastic modulus (Eq. (1)) and hardness (Eq. (2)) using the Oliver‐Pharr method, where S is the slope of the 95%–40% region of the unloading curve, β is a geometric factor derived from the shape of the indenter probe, A is the contact area of the probe with the specimen surface, and P_max_ is the maximum load on the load–displacement curve.^(^ ^24^ ^)^

(1) $$E_{r}=\frac{1}{\beta }\frac{S\sqrt{\pi }}{2\sqrt{A}}\;$$

(2) $$H=\frac{P_{\max}}{A}\;$$

A second section from a random subset of *n* = 6 femurs per group (adjacent to the previous) was used for colocalized Raman spectroscopy and nanoindentation in osteocyte perilacunar bone regions. Six osteocytes per sample within the posterolateral region were selected, and points were chosen at 1, 3, 5, and 7 μm from the lacunar wall in four directions (Fig. 2*A*, lower panel). This subset was used to balance the number of samples with the number of analyzed osteocyte perilacunar regions and limit the amount of time each sample underwent testing while providing 144 data points per group per lacunar distance. Colocalized Raman spectroscopy and nanoindentation were performed as described above. In approaching the perilacunar data analysis, we considered that there may be a heterogeneous response to high PTH and PTH lowering among osteocyte perilacunar regions. Therefore, we plotted the resulting data as histograms instead of averaging all data points together to bring out potential differences in lacunar properties between osteocyte regions.

### Quantitative backscatter electron imaging

Following nanoindentation, the *n* = 6 samples used for perilacunar measurements were removed from their slides and dehydrated using a vacuum pump for 48 hours. Specimens underwent carbon coating using carbon evaporation in preparation for backscatter electron imaging on a scanning electron microscope (JEOL 7800F; Akishima, Tokyo, Japan), a technique that has been validated for measuring mineral density distribution in bone.^(^ ^25^ ^)^ Regions of newly formed bone were found using image superposition with fluorescent microscopy images. Samples were imaged at 10 kV power and 73 μA beam current using a BED‐C retractable backscatter electron detector (JEOL, Akishima, Tokyo, Japan). Imaging began when the chamber pressure was <9.6E−5 Pa to reduce charging effects. Completed images were exported as .bmp bitmap files for analysis. Contrast adjustment was performed using backscatter images of standards with known atomic number: aluminum (*Z* = 13) and carbon (*Z* = 6). Brightness and contrast adjustments were performed by obtaining a gray level for aluminum of 225 ± 1 and carbon of 25 ± 1. Histograms of newly formed bone regions were created using ImageJ software (NIH, Bethesda, MD, USA; https://imagej.nih.gov/ij/) and were used to calculate bone mineral density distributions (BMDDs). Distributions were analyzed for mean calcium content across the region of interest (Ca_Mean_), peak, or the most frequent calcium content in the region of interest (Ca_Peak_), and calcium content heterogeneity using the full width at half maximum of the BMDD (Ca_Width_).

### Statistical analysis

Periosteal data (Raman spectroscopy, nanoindentation, and quantitative backscatter electron imaging [QBEI]) were checked for normality using a Shapiro‐Wilk test. Data that passed normality were analyzed by one‐way ANOVA with post hoc Tukey's test to determine differences in composition and mechanical properties between the three animal groups. Ca_Width_ was the only parameter that did not pass normality within all groups and was analyzed using a Kruskal‐Wallis test. All periosteal data was analyzed using Prism 9.3.1 (GraphPad Software, Inc., La Jolla, CA, USA). Perilacunar cumulative distribution data for Raman spectroscopy and nanoindentation were analyzed by Anderson‐Darling (more sensitive to the tails of the distribution) and Kolmogorov‐Smirnov (more sensitive to the bulk of the distribution) tests with a Bonferroni correction (significance set at *p* = 0.0167) to determine differences in property distributions in the perilacunar region using a custom MATLAB script. We found universal agreement between these two tests for Raman and nanoindentation data. Throughout the results, data will be described as “right‐shifted” if the distribution was found to have significantly higher values for a given property.

## Results

### CKD and KP treatment alter periosteal bone matrix composition and mechanics

**Fig. 3:**
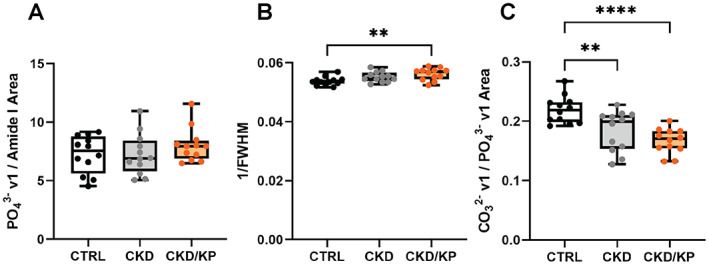
KP treatment increases periosteal mineral crystallinity and lowers type B carbonate substitution. Mineral‐to‐matrix ratio was not different between groups (*A*). Mineral crystallinity was significantly higher in CKD/KP versus CTRL (*B*). Type B carbonate substitution was significantly lower in CKD and CKD/KP versus CTRL (*C*). Data are shown as mean ± SD and analyzed by one‐way ANOVA. If *p* < 0.05, Tukey's range test was performed: **p < 0.01; ****p < 0.0001.

**Fig. 4:**
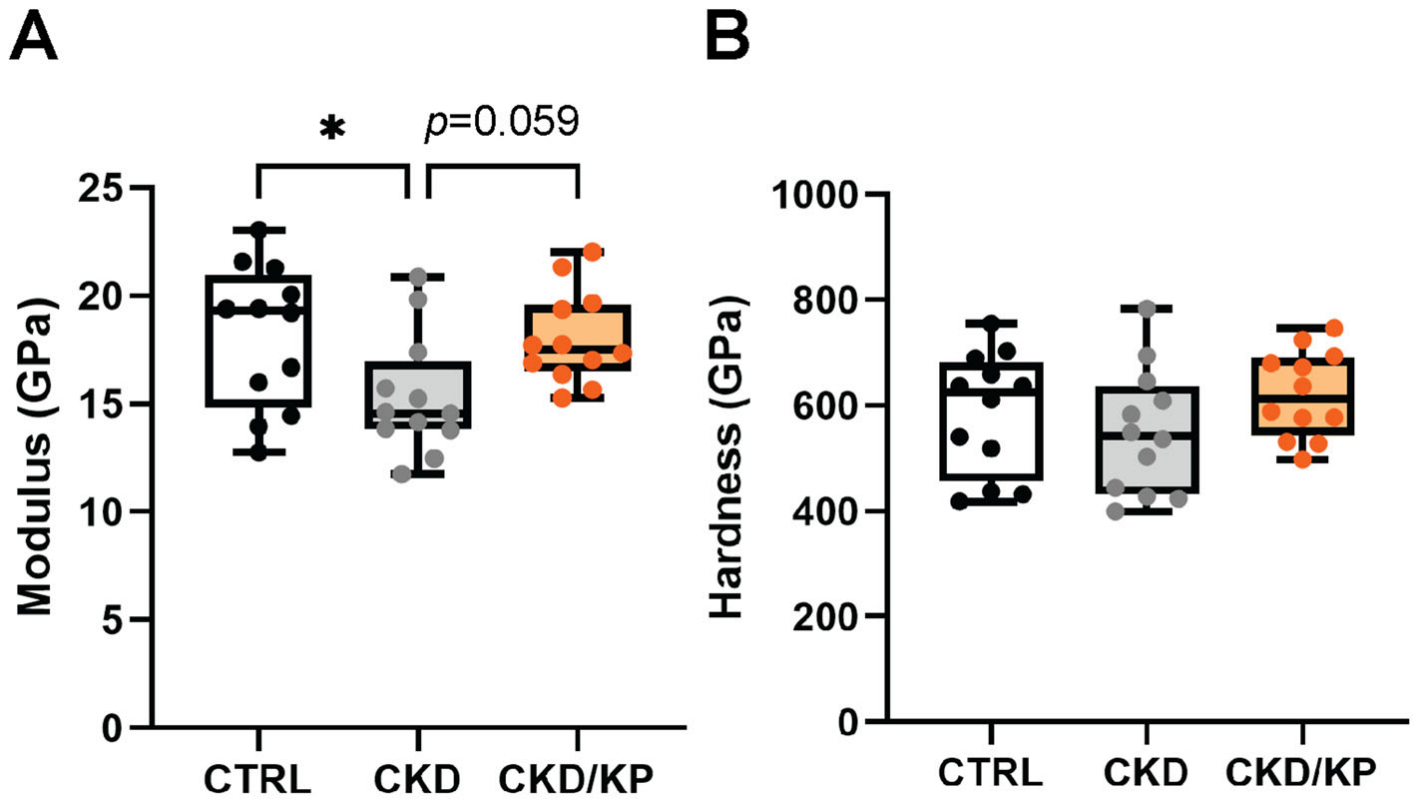
KP treatment maintains periosteal reduced modulus at CTRL levels. Reduced modulus was significantly lower in CKD versus CTRL (*A*). CKD/KP increased reduced modulus versus CKD but was not significantly different from CKD or CTRL (*A*). Tissue hardness was not different between groups (*B*). Data are shown as mean ± SD and analyzed by one‐way ANOVA. If *p* < 0.05, Tukey's range test was performed: *p < 0.05.

As reported, PTH was 3.8‐fold higher in CKD animals versus CTRL and was 1.9‐fold lower in KP‐treated rats versus CKD.^(^ ^22^ ^)^ There were no differences in calcium levels. Raman spectroscopy in new periosteal bone, taken as the region between calcein labels, revealed no differences in mineral‐to‐matrix ratio (MMR) between the three groups (Fig. 3*A*). Mineral crystallinity was significantly higher in CKD/KP animals versus CTRL (Fig. 3*B*). Type B carbonate substitution was significantly lower in CKD and CKD/KP animals versus CTRL (Fig. 3*C*). Nanoindentation in this periosteal region showed that reduced elastic modulus was significantly lower in CKD versus CTRL (Fig. 4*A*). Reduced modulus was higher in CKD/KP versus CKD, but failed to reach significance (*p* = 0.059, Fig. 4*A*). Tissue hardness was not different between the three groups (Fig. 4*B*).

### KP treatment does not alter calcium content in new periosteal bone

**Fig. 5:**
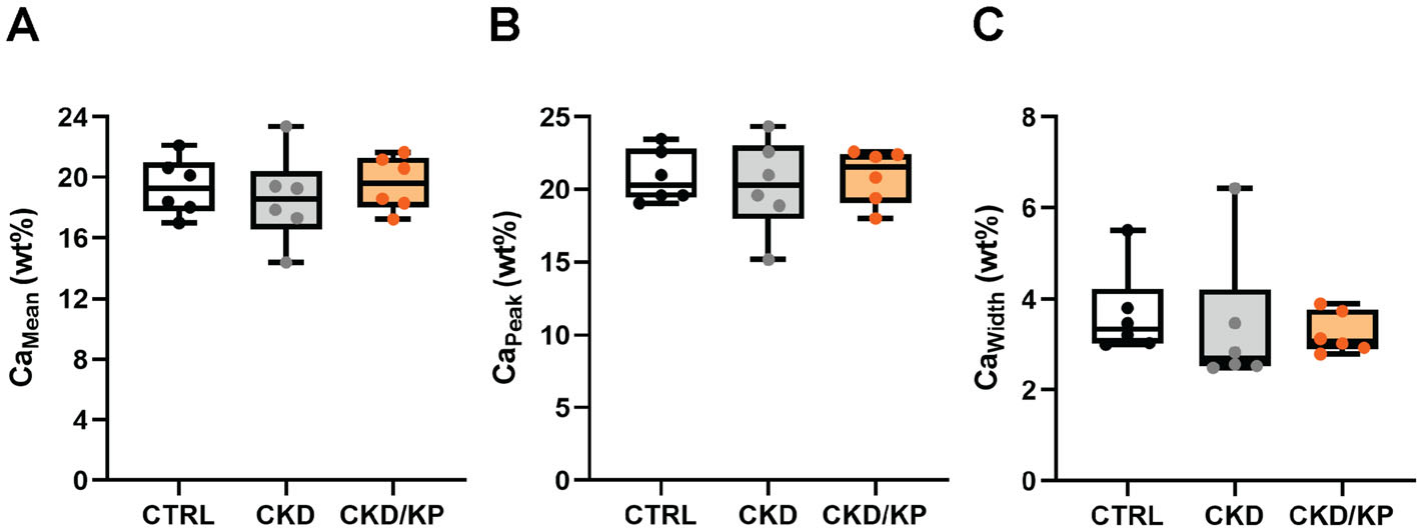
CKD and KP treatment do not alter periosteal calcium content. No differences between groups were found in mean or peak calcium content or in calcium distribution width/heterogeneity (*A*–*C*). Data are shown as mean ± SD. Ca_Mean_ and Ca_Peak_ were analyzed by one‐way ANOVA and Ca_Width_ was analyzed using a Kruskal‐Wallis test.

Bone mineral density distributions of newly formed bone regions were used to determine whether CKD or KP treatment alter calcium content in age‐matched regions of bone. No differences were found in Ca_Mean_, Ca_Peak_, or Ca_Width_ between groups (Fig. 5*A*–*C*), indicating that changes in calcium, a metric that is not detected by Raman spectroscopy, was likely not responsible for the altered material properties seen using nanoindentation.

### CKD and KP treatment alter perilacunar bone matrix composition and mechanics in a spatially dependent manner

**Fig. 6:**
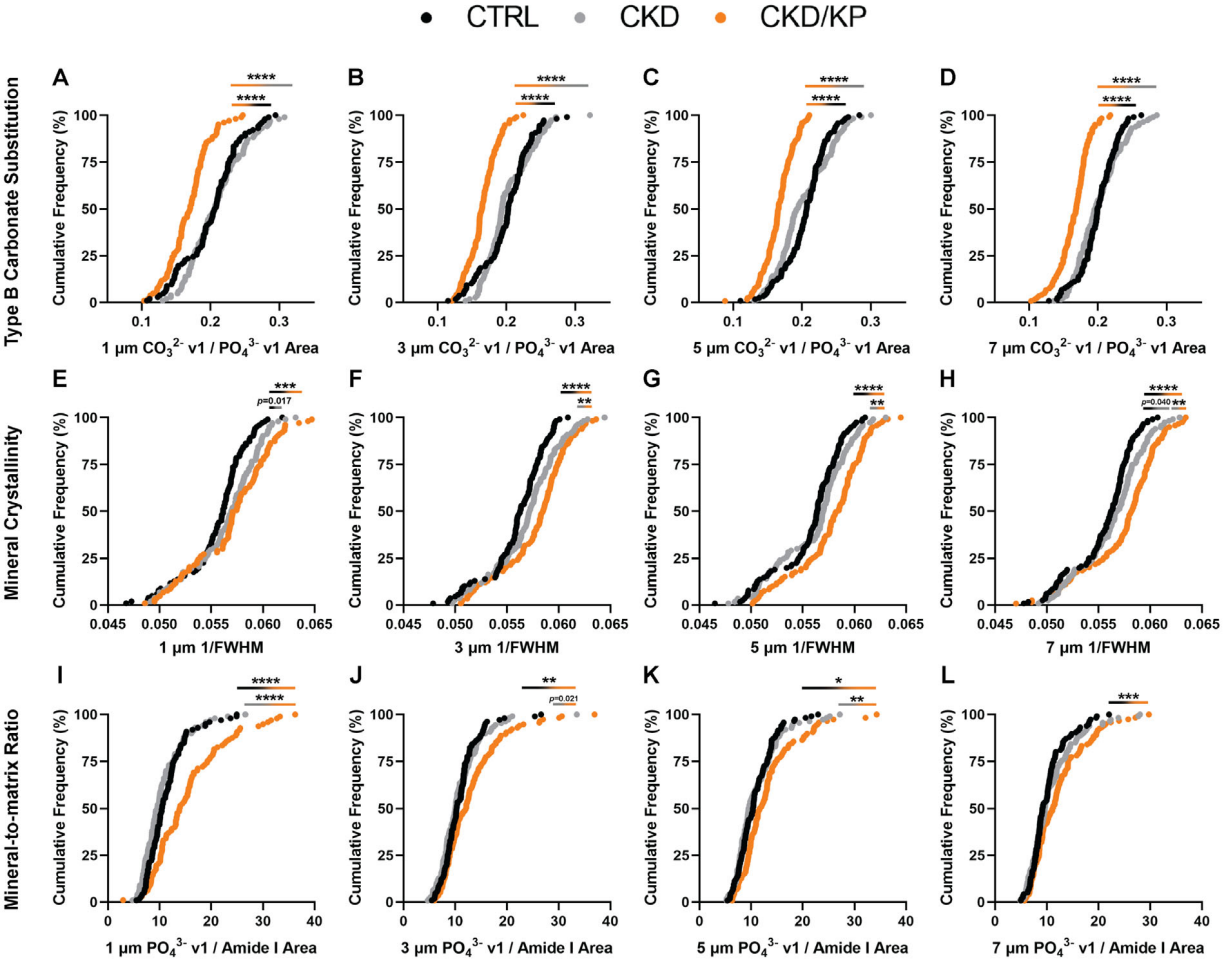
KP treatment lowers perilacunar carbonate substitution while increasing crystallinity and mineral‐to‐matrix ratio. Type B carbonate substitution was right‐shifted in CTRL and CKD versus CKD/KP at all distances (*A*–*D*). Mineral crystallinity was right‐shifted in CKD/KP versus CTRL at all distances and versus CKD at 3, 5, and 7 μm (*E*–*H*). Mineral‐to‐matrix ratio was right‐shifted in CKD/KP versus CTRL at all distances and versus CKD at 1 and 5 μm (*I*–*L*). Data were analyzed by Anderson‐Darling and Kolmogorov‐Smirnov tests with a Bonferroni correction: *p < 0.0167; **p < 0.01; ***p < 0.001; ****p < 0.0001.

To determine the large‐scale distribution of compositional properties in the perilacunar region, we analyzed histograms incorporating all Raman signatures collected, separated by lacunar distance (144 measurements per lacunar distance per group). Type B carbonate substitution distribution was significantly right‐shifted in CTRL and CKD versus CKD/KP at all distances (Fig. 6*A*–*D*). Mineral crystallinity was significantly right‐shifted in CKD/KP versus CTRL at all distances and versus CKD at 3, 5, and 7 μm (Fig. 6*E*–*H*). Finally, we found that MMR distribution was significantly right‐shifted in CKD/KP versus CTRL at all locations, and versus CKD at 1 and 5 μm from the lacunar wall (Fig. 6*I*–*L*). MMR in CKD/KP versus CKD at 3 μm had a right‐shift trend but did not pass our modified *p* value threshold from the Bonferroni correction.

**Fig. 7:**
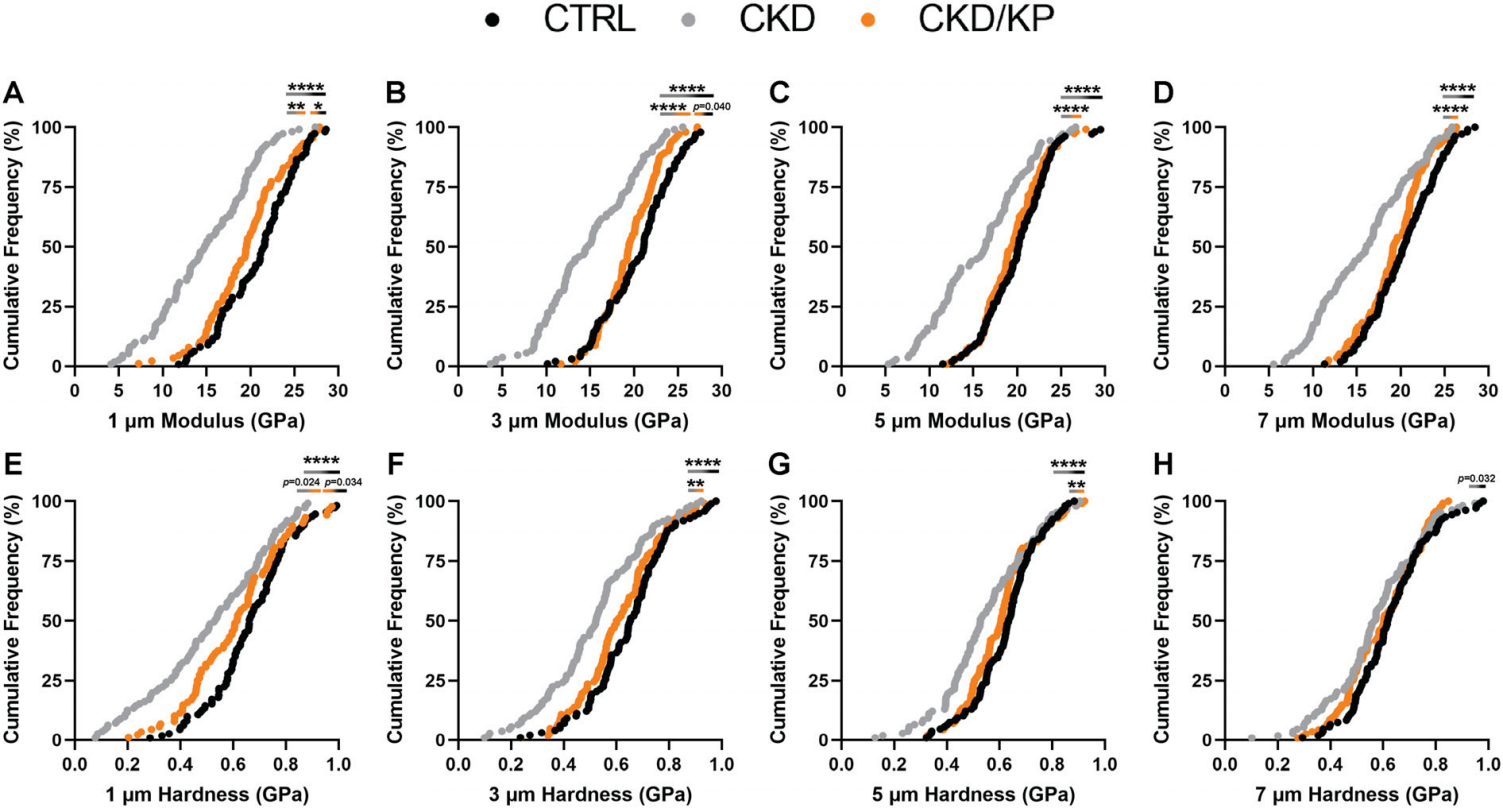
KP treatment improves material property distributions in perilacunar bone. Reduced modulus was significantly right‐shifted in CKD/KP and CTRL versus CKD at all distances (*A*–*D*). CTRL reduced modulus was significantly right‐shifted versus CKD/KP at 1 μm (*A*). Hardness was significantly right‐shifted in CKD/KP versus CKD at 3 and 5 μm and in CTRL versus CKD at 1, 3, and 5 μm (*E*–*H*). Data were analyzed by Anderson‐Darling and Kolmogorov‐Smirnov tests with a Bonferroni correction: *p < 0.0167; **p < 0.01; ***p < 0.001; ****p < 0.0001.

Nanoindentation in the perilacunar region demonstrated a significantly right‐shifted reduced modulus distribution in CKD/KP and CTRL versus CKD at all distances (Fig. 7*A*–*D*). Reduced modulus was also significantly right‐shifted in CTRL versus CKD/KP at 1 μm but was not different at 3, 5, or 7 μm (Fig. 7*A*–*D*). For tissue hardness, distributions were significantly right‐shifted in CKD/KP versus CKD at 3, and 5 μm (Fig. 7*E*–*G*) with a higher trend at 1 μm. CTRL distributions were significantly right‐shifted versus CKD at 1, 3, and 5 μm and trended higher versus CKD/KP at 1 μm (Fig. 7*E*–*H*).

## Discussion

In this study, we determined that early‐stage CKD and calcimimetic intervention alter matrix‐level bone composition and material properties in newly formed periosteal bone and in the osteocyte perilacunar region. CKD lowered type B carbonate substitution versus CTRL in new periosteal bone and lowered reduced modulus at all locations in perilacunar bone. CKD also lowered hardness up to 5 μm from the lacunar wall versus CTRL. KP treatment increased mineral crystallinity in new periosteal bone, lowered type B carbonate substitution in perilacunar bone, and generally increased reduced modulus and hardness in perilacunar bone versus CKD. These data support the idea that CKD has detrimental effects on the bone matrix that may occur prior to clinically visible signs of bone disease (eg, prior to the onset of increased cortical porosity) and that calcimimetics may help mitigate or prevent these alterations.

Male Cy/+ rats represent a robust and gradually progressive model of CKD‐MBD, undergoing biochemical alterations related to mineral homeostasis, aortic calcification, culminating in structural bone alterations including cortical thinning and pore formation at 35 weeks of age. Recently, our group analyzed a cohort of 28‐week‐old CKD rats and found that despite having low kidney function and high PTH versus control rats, microcomputed tomography detected no structural and limited mechanical changes in the long bones.^(^ ^22^ ^)^ Therefore, we hypothesized that performing Raman spectroscopy and nanoindentation on 28‐week‐old specimens may reveal changes in the bone matrix that precede pore formation. Although tissue‐level analyses have been performed in rodent models of CKD, including the Cy/+ rat, controlling for tissue age by injecting calcein double labels is not common in published data. Indeed, Raman spectroscopy and nanoindentation of 35‐week old Cy/+ rats without controlling for the tissue age of the selected regions has previously revealed no differences in tissue‐level composition or mechanics versus CTRL rats.^(^ ^26^ ^)^ Given the progressive nature of bone deterioration in CKD, we hypothesize that controlling for tissue age was necessary to detect differences in compositional and material properties between groups in this study.

Numerous published studies perform Raman spectroscopy and nanoindentation on fixed and/or dehydrated specimens, often using matrix‐infiltrating poly (methyl methacrylate) to embed the bones.^(^ ^27^, ^28^ ^)^ Recent research suggests that a loss of bone water during sample dehydration increases stiffness and lowers postyield properties such as toughness and can mask disease‐induced alterations to bone matrix quality and material properties.^(^ ^29^, ^30^ ^)^ Postyield properties are critical to fracture resistance in bone, as material toughening mechanisms are necessary to prevent rapid crack propagation and mechanical failure, a feature that is partially portrayed in tissue hardness data.^(^ ^31^ ^)^ Embedding media and sample fixation/dehydration have also have significant impacts on Raman spectral parameters, as the infiltrating media create overlapping bands in the Raman signature.^(^ ^32^ ^)^ To the best of our knowledge, we are among the first to use fresh specimens while preserving hydration by humidifying the specimen chamber during Raman spectroscopy and submerging the samples in PBS during nanoindentation. We hypothesize that our method reduced the heterogeneity brought by averaging data points from all bone regions, allowing for a focused analysis on skeletal properties in regions formed under high PTH, with or without treatment. Taken together, our use of age‐matched regions in fresh, hydrated samples preserved the native tissue state and the intrinsic compositional and mechanical features of the bone.

Bone mineral is a carbonated hydroxyapatite where substitution of phosphate for carbonate is driven by the similar size and charge between the two ionic compounds. We found significantly less type B carbonate substitution in new periosteal bone in CKD animals versus CTRL and an upward trend in crystallinity in CKD versus CTRL (Fig. 3*B*,*C*). Previous studies suggest that carbonate substitution lowers mineral crystallinity as carbonate dilates the *c*‐axis and shrinks the *a*‐axis of the mineral crystal, indicating that the bond stretching induced by the presence of carbonate may not have been enough to measure a change in crystallinity using Raman.^(^ ^33^ ^)^ However, we did observe a larger magnitude difference in carbonate substitution in KP‐treated rats versus CTRL, which may have driven the significantly higher crystallinity observed in CKD/KP versus CTRL.

In terms of material properties, there was no difference in reduced modulus in CKD/KP versus CTRL (Fig. 4*A*). Reduced elastic modulus is a tissue‐level measure of material stiffness (resistance to elastic deformation) and is positively correlated with higher mineralization and crystallinity.^(^ ^34^ ^)^ Although the increase in crystallinity likely contributed to the higher reduced modulus seen in CKD/KP versus CKD, the lack of change in MMR (Fig. 3*A*) or BMDD (Fig. 5) versus CTRL may have prevented the reduced modulus from increasing beyond that of CTRL rats. Finally, we found that reduced modulus was significantly lower in CKD versus CTRL despite the lack of differences in MMR, BMDD, and crystallinity. Although surprising, these data may indicate that CKD bone has further structural deficits such as increased nanopores that are not measurable via microcomputed tomography. However, these data support the idea that higher mineral quality contributes to higher reduced modulus in KP‐treated rats.

PTH signaling is essential for triggering matrix remodeling by osteocytes, a process that is critical in mediating calcium release and restoration in lactation and weaning, respectively.^(^ ^20^ ^)^ Although this phenomenon has not been explored in CKD where high PTH is common, we hypothesized that osteocyte interactions with the bone matrix could contribute to the skeletal detriments in CKD, and that altered matrix composition and material properties can be detected in the perilacunar region when PTH is high but porosity is not evident. Notably, we found a higher MMR at 1 and 5 μm from the lacunar wall in CKD/KP versus CKD, a higher trend at 3 μm, but no difference at 7 μm (Fig. 6*I*–*L*). MMR is reflective of the relative mineralization of the tissue, indicating that calcimimetics increase relative tissue mineralization in CKD up to 5 μm from the osteocyte lacuna. MMR was also higher in CKD/KP versus CTRL at all distances. Taken together, these data indicate that while CKD did not change relative mineralization of the perilacunar bone, calcimimetics are capable of increasing mineralization in the perilacunar region up to 5 μm from the lacunar wall.

Interestingly, the trends in matrix composition in CKD/KP versus CKD are paralleled by colocalized mechanical data obtained from perilacunar nanoindentation. Reduced elastic modulus was higher in CKD/KP versus CKD at all perilacunar distances and modulus distributions in CKD/KP were not different from CTRL at 3, 5 and 7 μm (Fig. 7*A*–*D*). MMR was significantly higher or trended higher at all distances except 7 μm and crystallinity was higher at all but 1 μm in CKD/KP versus CKD. In particular, the MMR data points to a specific region around osteocytes that can undergo further mineralization by lowering PTH in CKD. Although the changes in MMR and crystallinity do not completely overlap, one or the other is increased at each perilacunar distance, which is likely responsible for the increased modulus seen at all locations. Curiously, despite the higher MMR and crystallinity in CKD/KP versus CTRL, reduced modulus was not different between these groups. Alongside these compositional changes, type B carbonate substitution was lower at all perilacunar distances in CKD/KP versus CTRL. Although type B carbonate substitution is inversely correlated with crystallinity as previously discussed, studies in rat femurs have shown that pathological accumulation of carbonate can embrittle the bone by inducing local strains and altering crystal growth.^(^ ^35^ ^)^ Therefore, we hypothesize that lower type B carbonate substitution induced by KP treatment balanced the increased MMR, resulting in bone tissue with similar material properties to healthy bone.

Although several compositional differences were found between CKD/KP and CKD in the perilacunar region, the only difference detected in CKD versus CTRL was a right‐shift trend in mineral crystallinity at 1 and 7 μm from the lacunar wall, whereas mechanical properties were lower at all perilacunar distances in CKD versus CTRL (Figs. 6 and 7). This likely indicates that CKD causes material alterations that were not detected by our methods, such as disrupted collagen quality through the formation of advanced glycation end products, which are formed in CKD due to increased oxidative stress and inflammation.^(^ ^36^, ^37^ ^)^ We hypothesize that the high crystallinity near the lacunae may be caused by high serum phosphorus in CKD, which may be incorporated by the osteocyte into newly formed, highly crystalline bone. However, additional detriments must exist that prevent this change from resulting in a higher reduced modulus in the region nearest the osteocyte lacuna.

Tissue hardness was also higher in CKD/KP versus CKD perilacunar bone up to 5 μm from the lacunar wall (Fig. 7*E*–*H*). Hardness is reflective of the ability of a material to resist plastic/permanent deformation, a property that depends on the organic components of the bone matrix including collagen and water and has been correlated with higher mineralization.^(^ ^31^ ^)^ Although higher MMR and crystallinity typically increase reduced elastic modulus and lower hardness, it is possible that collagen content/quality and/or bound water were also higher in these regions to compensate for the increased mineralization. Future studies specifically interrogating the role of collagen quality and hydration in bone material properties in CKD are warranted.

In conclusion, CKD and calcimimetic treatment altered bone matrix composition and mechanical properties in animals representing approximately stage 4 CKD despite the lack of overt bone deterioration at this time point. These findings may contribute to the increased fracture resistance postulated by analysis of the EVOLVE trial, demonstrating a crucial parallel between microscale data and clinical findings. Although the notion that microscale bone properties determine the macroscale material properties of the bone is not new, our study is among the first to analyze matrix composition and mechanics in colocalized, tissue age‐matched regions of hydrated bone. This opens the possibility to use micromechanical testing to analyze viscoelastic properties, where maintaining native tissue hydration is critical. Future studies will also examine whether initiating treatment at this time point improves skeletal microarchitecture and mechanical properties at a later time point representing ESKD, which may point to the efficacy of lowering PTH earlier in the disease course of CKD.

## Author Contributions

**John G Damrath:** Conceptualization; data curation; formal analysis; funding acquisition; investigation; methodology; visualization; writing – original draft; writing – review and editing. **Sharon M Moe:** Conceptualization; funding acquisition; methodology; resources; supervision; writing – review and editing. **Joseph M Wallace:** Conceptualization; formal analysis; funding acquisition; methodology; resources; supervision; writing – review and editing.

## Conflicts of Interest

JGD, JMW: None. SMM: Scientific consulting for Amgen.

### Peer Review

The peer review history for this article is available at https://publons.com/publon/10.1002/jbmr.4574.

## Data Availability

The data that support the findings of this study are available from the corresponding author upon reasonable request.

## References

[1] Moe SM , Nickolas TL . Fractures in patients with CKD: time for action. Clin J Am Soc Nephrol. 2016;11(11):1929‐1931. 27797903 10.2215/CJN.09500916 PMC5108207

[2] Centers for Disease Control and Prevention . Chronic kidney disease in the United States, 2021. Atlanta, GA: US Department of Health and Human Services, Centers for Disease Control and Prevention; 2021. https://www.cdc.gov/kidneydisease/publications-resources/ckd-national-facts.html. Accessed May 27, 2022.

[3] Moe S , Drüeke T , Cunningham J , et al. Definition, evaluation, and classification of renal osteodystrophy: a position statement from Kidney Disease: Improving Global Outcomes (KDIGO). Kidney Int. 2006;69(11):1945‐1953. 16641930 10.1038/sj.ki.5000414

[4] Nickolas TL , Stein EM , Dworakowski E , et al. Rapid cortical bone loss in patients with chronic kidney disease. J Bone Miner Res. 2013;28(8):1811‐1820. 23456850 10.1002/jbmr.1916 PMC3720694

[5] Coco M , Rush H . Increased incidence of hip fractures in dialysis patients with low serum parathyroid hormone. Am J Kidney Dis. 2000;36(6):1115‐1121. 11096034 10.1053/ajkd.2000.19812

[6] Moe SM , Abdalla S , Chertow GM , et al. Effects of Cinacalcet on fracture events in patients receiving hemodialysis: the EVOLVE trial. J Am Soc Nephrol. 2015;26(6):1466‐1475. 25505257 10.1681/ASN.2014040414 PMC4446874

[7] Metzger CE , Swallow EA , Stacy AJ , et al. Reversing cortical porosity: cortical pore infilling in preclinical models of chronic kidney disease. Bone. 2021;143:115632. 32927105 10.1016/j.bone.2020.115632 PMC7770083

[8] Hernandez CJ , van der Meulen MC . Understanding bone strength is not enough. J Bone Miner Res. 2017;32(6):1157‐1162. 28067411 10.1002/jbmr.3078 PMC5466476

[9] Morris MD , Mandair GS . Raman assessment of bone quality. Clin Orthop Relat Res. 2011;469(8):2160‐2169. 21116756 10.1007/s11999-010-1692-y PMC3126952

[10] Paschalis EP , Mendelsohn R , Boskey AL . Infrared assessment of bone quality: a review. Clin Orthop Relat Res. 2011;469(8):2170‐2178. 21210314 10.1007/s11999-010-1751-4 PMC3126953

[11] Hoffler CE , Guo XE , Zysset PK , Goldstein SA . An application of nanoindentation technique to measure bone tissue lamellae properties. J Biomech Eng. 2005;127(7):1046‐1053. 16502646 10.1115/1.2073671

[12] Donnelly E , Boskey AL , Baker SP , van der Meulen MC . Effects of tissue age on bone tissue material composition and nanomechanical properties in the rat cortex. J Biomed Mater Res A. 2010;92(3):1048‐1056. 19301272 10.1002/jbm.a.32442 PMC4160143

[13] Bellido T , Ali AA , Gubrij I , et al. Chronic elevation of parathyroid hormone in mice reduces expression of sclerostin by osteocytes: a novel mechanism for hormonal control of osteoblastogenesis. Endocrinology. 2005;146(11):4577‐4583. 16081646 10.1210/en.2005-0239

[14] Xiong J , Piemontese M , Thostenson JD , Weinstein RS , Manolagas SC , O'Brien CA . Osteocyte‐derived RANKL is a critical mediator of the increased bone resorption caused by dietary calcium deficiency. Bone. 2014;66:146‐154. 24933342 10.1016/j.bone.2014.06.006 PMC4125539

[15] Gardinier JD , Al‐Omaishi S , Morris MD , Kohn DH . PTH signaling mediates perilacunar remodeling during exercise. Matrix Biol. 2016;52‐54:162‐175. 10.1016/j.matbio.2016.02.010 PMC4875803 26924474

[16] Qing H , Ardeshirpour L , Pajevic PD , et al. Demonstration of osteocytic perilacunar/canalicular remodeling in mice during lactation. J Bone Miner Res. 2012;27(5):1018‐1029. 22308018 10.1002/jbmr.1567 PMC3770147

[17] Dole NS , Mazur CM , Acevedo C , et al. Osteocyte‐intrinsic TGF‐β signaling regulates bone quality through perilacunar/canalicular remodeling. Cell Rep. 2017;21(9):2585‐2596. 29186693 10.1016/j.celrep.2017.10.115 PMC6014615

[18] Creecy A , Damrath JG , Wallace JM . Control of bone matrix properties by osteocytes. Front Endocrinol. 2020;11:578477. 10.3389/fendo.2020.578477 PMC7848033 33537002

[19] Tanaka T , Hoshijima M , Sunaga J , et al. Analysis of Ca(2+) response of osteocyte network by three‐dimensional time‐lapse imaging in living bone. J Bone Miner Metab. 2018;36(5):519‐528. 29027020 10.1007/s00774-017-0868-x

[20] Jähn K , Kelkar S , Zhao H , et al. Osteocytes acidify their microenvironment in response to PTHrP in vitro and in lactating mice in vivo. J Bone Miner Res. 2017;32(8):1761‐1772. 28470757 10.1002/jbmr.3167 PMC5550338

[21] Moe SM , Chen NX , Seifert MF , et al. A rat model of chronic kidney disease‐mineral bone disorder. Kidney Int. 2009;75(2):176‐184. 18800026 10.1038/ki.2008.456 PMC2716076

[22] Damrath JG , Chen NX , Metzger CE , et al. Non‐additive effects of combined NOX1/4 inhibition and calcimimetic treatment on a rat model of chronic kidney disease‐mineral and bone disorder (CKD‐MBD). JBMR Plus. 2022;6(3):e10600. 35309859 10.1002/jbm4.10600 PMC8914155

[23] Berman AG , Damrath JG , Hatch J , et al. Effects of Raloxifene and tibial loading on bone mass and mechanics in male and female mice. Connect Tissue Res. 2022;63(1):3‐15. 33427519 10.1080/03008207.2020.1865938 PMC8272732

[24] Oliver WC , Pharr GM . An improved technique for determining hardness and elastic modulus using load and displacement sensing indentation experiments. J Mater Res. 1992;7(6):1564‐1583.

[25] Roschger P , Fratzl P , Eschberger J , Klaushofer K . Validation of quantitative backscattered electron imaging for the measurement of mineral density distribution in human bone biopsies. Bone. 1998;23(4):319‐326. 9763143 10.1016/s8756-3282(98)00112-4

[26] Newman CL , Moe SM , Chen NX , et al. Cortical bone mechanical properties are altered in an animal model of progressive chronic kidney disease. PLoS One. 2014;9(6):e99262. 24911162 10.1371/journal.pone.0099262 PMC4049798

[27] Heveran CM , Schurman CA , Acevedo C , et al. Chronic kidney disease and aging differentially diminish bone material and microarchitecture in C57Bl/6 mice. Bone. 2019;127:91‐103. 31055118 10.1016/j.bone.2019.04.019 PMC6760860

[28] Gardinier JD , Al‐Omaishi S , Rostami N , Morris MD , Kohn DH . Examining the influence of PTH(1‐34) on tissue strength and composition. Bone. 2018;117:130‐137. 30261327 10.1016/j.bone.2018.09.019 PMC6202137

[29] Nyman JS , Roy A , Shen X , Acuna RL , Tyler JH , Wang X . The influence of water removal on the strength and toughness of cortical bone. J Biomech. 2006;39(5):931‐938. 16488231 10.1016/j.jbiomech.2005.01.012 PMC1941695

[30] Vesper EO , Hammond MA , Allen MR , Wallace JM . Even with rehydration, preservation in ethanol influences the mechanical properties of bone and how bone responds to experimental manipulation. Bone. 2017;97:49‐53. 28057526 10.1016/j.bone.2017.01.001 PMC5367983

[31] Ibrahim A , Magliulo N , Groben J , Padilla A , Akbik F , Abdel HZ . Hardness, an important indicator of bone quality, and the role of collagen in bone hardness. J Funct Biomater. 2020;11(4):85. 10.3390/jfb11040085 PMC7712352 33271801

[32] Yeni YN , Yerramshetty J , Akkus O , Pechey C , Les CM . Effect of fixation and embedding on Raman spectroscopic analysis of bone tissue. Calcif Tissue Int. 2006;78(6):363‐371. 16830201 10.1007/s00223-005-0301-7

[33] Fleet ME , Liu X , King PL . Accommodation of the carbonate ion in apatite: an FTIR and X‐ray structure study of crystals synthesized at 2–4 GPa. Am Mineral. 2004;89(10):1422‐1432.

[34] Yerramshetty JS , Akkus O . The associations between mineral crystallinity and the mechanical properties of human cortical bone. Bone. 2008;42(3):476‐482. 18187375 10.1016/j.bone.2007.12.001

[35] Akkus O , Adar F , Schaffler MB . Age‐related changes in physicochemical properties of mineral crystals are related to impaired mechanical function of cortical bone. Bone. 2004;34(3):443‐453. 15003792 10.1016/j.bone.2003.11.003

[36] Rapa SF , Di Iorio BR , Campiglia P , Heidland A , Marzocco S . Inflammation and oxidative stress in chronic kidney disease‐potential therapeutic role of minerals, vitamins and plant‐derived metabolites. Int J Mol Sci. 2019;21(1):263. 10.3390/ijms21010263 PMC6981831 31906008

[37] Damrath JG , Creecy A , Wallace JM , Moe SM . The impact of advanced glycation end products on bone properties in chronic kidney disease. Curr Opin Nephrol Hypertens. 2021;30(4):411‐417. 33928911 10.1097/MNH.0000000000000713 PMC8154706

